# Genome-wide association study revealed genetic variations of ABA sensitivity controlled by multiple stress-related genes in rice

**DOI:** 10.1007/s44154-021-00011-4

**Published:** 2021-10-07

**Authors:** Lei Peng, Tingting Xie, Zilong Guo, Xiaokai Li, Yu Chang, Haifu Tu, Shengchang Wang, Nai Wu, Yilong Yao, Lizhong Xiong

**Affiliations:** National Key Laboratory of Crop Genetic Improvement, Hubei Hongshan Laboratory, Huazhong Agricultural University, Wuhan, 430070 China

**Keywords:** ABA sensitivity, Seed germination, Stress response, Rice

## Abstract

Abscisic acid (ABA) is a critical phytohormone that regulates multiple physiological processes including plant growth and stress tolerance. The core ABA signaling pathway has been well established, but genetic variations mediating ABA responses remain largely unknown. In this study, we performed genome-wide association study (GWAS) to identify loci and genes associated with ABA sensitivity (reflected by seed germination inhibition by ABA) in a panel of 425 rice accessions. The seed germination assay revealed that *Aus* and *indica* rice had stronger ABA sensitivity than *japonica* rice. A total of 48 non-redundant association loci were detected in the *indica* subpopulation and whole population, and 386 genes in these loci were responsive to ABA or abiotic stresses. Eight association loci were overlapped with previously reported loci for yield under drought stress or for drought-indicative image traits. Haplotype analyses of important candidate genes such as *OsSAPK6*, a key component in the ABA signaling core, were performed to identify key SNPs/InDels that may affect gene functions through promoter activity regulation, amino acid variation, or gene splicing. These results provide insights into the genetic basis of ABA sensitivity related to stress responses.

**Supplementary Information:**

The online version contains supplementary material available at 10.1007/s44154-021-00011-4.

## Introduction

Abscisic acid (ABA) is an important stress hormone for the responses and adaptation of plants to abiotic stresses by controlling multiple processes of plant growth and development. ABA plays a crucial role in various physiological processes, such as stomatal closure, cuticular wax accumulation, leaf senescence, bud dormancy, growth inhibition, osmotic regulation, and seed germination (reviewed in (Chen et al. 2020)). Due to the discovery of ABA receptors, our understanding of the ABA signaling pathway has been largely deepened (Ma et al. 2009; Park et al. 2009). ABA signaling core, including ABA receptor PYRABACTIN RESISTANCE/PYRABACTIN RESISTANCE-LIKE/REGULATORY COMPONENTS OF THE ABSCISIC ACID RECEPTOR (PYR/PYL/RCAR), clade-A type-2C protein phosphatase (PP2C), and SNF1-related protein kinase 2 (SnRK2), have been well-known for their canonical molecular mechanisms in multiple ABA-induced plant physiological processes (Chen et al. 2020; Cutler et al. 2010). When there is no ABA, PP2Cs dephosphorylate SnRK2 kinases and inhibit their activities (Umezawa et al. 2009; Vlad et al. 2009). When ABA is bound by its receptors PYR/PYL/RCAR, PP2Cs are inhibited by the receptors through direct binding, leading to activation of SnRK2 (Ma et al. 2009; Park et al. 2009; Tischer et al. 2017). Activated SnRK2s phosphorylate substrate proteins such as transcription factors including ABSCISIC ACID INSENSITIVE 5 (ABI5) which subsequently regulate arrays of genes related to stress responses (Fujii and Zhu 2009).

Diverse proteins are involved in ABA signaling by regulating activity of the ABA signaling core components. ABA receptor PYL4 can be phosphorylated and inhibited by TARGET OF RAPAMYCIN (TOR) kinase, which is in turn inhibited by ABA through SnRK2-mediated protein phosphorylation of the REGULATORY-ASSOCIATED PROTEIN OF TOR 1B (RAPTOR1B) (Wang et al. 2018a). By contrast, PYR/PYL/RCAR members have been shown to be phosphorylated and activated by receptor-like cytoplasmic kinase CARK1 (Li et al. 2019; Zhang et al. 2018). According to a recent study, SnRK2.6 reactivation requires another essential signaling component belonging to B3 subfamily of Raf-like MAP kinase kinase kinase (MAPKKK) family (Takahashi et al. 2020). PP2C activity can be stabilized (Li and Liu 2012) or increased (Yu et al. 2012) by small GTP-binding protein Rho-like GTPase protein (ROP) family, which is a negative regulator of ABA signaling. ENHANCER OF ABA CO-RECEPTOR 1 (EAR1), another negative regulator of ABA, can enhance PP2C activities through direct interaction with N-terminal auto-inhibitory domain of PP2Cs (Wang et al. 2018b). In addition, several proteins including two U-Box E3 ligases (PUB12 and PUB13), two Ring-type E3 ligases (RGLG1 and RGLG5), and two adaptor proteins (DET1-AND DDB1-ASSOCIATED 1 [DDA1] and BTB/POZ AND MATH DOMAIN protein [BPM]) can regulate ABA signaling by degrading PYR/PYL/RCAR receptors or PP2Cs (Julian et al. 2019; Garcia-Leon et al. 2019; Wu et al. 2016; Kong et al. 2015; Irigoyen et al. 2014).

The timing of seed germination is crucial for plant growth in correct seasons. ABA inhibits germination but promotes seed maturation and dormancy (Koornneef et al. 2002). In 1990s, screening mutants of seed germination inhibition was an important approach for studying ABA signaling (Allen et al. 1999; Finkelstein and Lynch 2000; Giraudat et al. 1992; Huijser et al. 2000; Leung et al. 1994; Leung et al. 1997; Rodriguez et al. 1998). The seed germination-inhibited mutants were named ABI (ABA insensitive) mutants. *ABI1* and *ABI2*, encoding two PP2C protein phosphatases in the ABA signaling core, play essential roles in the regulation of seed germination (Leung et al. 1994; Leung et al. 1997). *ABI3*, *ABI4*, and *ABI5*, encoding transcription factors in the ABA signaling pathway, are also important to seed dormancy (Finkelstein and Lynch 2000; Giraudat et al. 1992; Huijser et al. 2000; Finkelstein et al. 1998). Besides the PYR/PYL/RCAR–PP2C–SnRK2 signaling core, an increasing number of proteins have been identified with roles in the complicated ABA molecular network for seed germination. These proteins include LEAFY COTYLEDON2 (LEC2), FUSCA3 (FUS3) (structurally related to ABI3/VP1), calcium-dependent protein kinases (CPKs), SnRK2-interacting calcium sensor (SCS), calcineurin B-like protein 9 (CBL9) and CBL-interacting protein kinase 3 (CIPK3), and late embryogenesis abundant (LEA) class proteins (reviewed in (Nakashima and Yamaguchi-Shinozaki 2013)).

Although the ABA signaling core have been intensively studied and widely accepted, the ABA signaling network and genetic variation of ABA response need to be further revealed, especially in economically important crops. In this study, genome-wide association study (GWAS) was used to identify genetic loci of ABA sensitivity, indicated by seed germination inhibition by ABA, in a global collection of 425 rice accessions. New association loci for ABA sensitivity and novel candidate genes involved in ABA signaling and/or stress tolerance were identified. Our results provided an overall scenario on the natural variation of ABA sensitivity in rice germplasms and identified numbers of significantly associated loci and candidate genes involved in the ABA signaling and multiple stress responses.

## Results

### Population structure and phenotypic variation in ABA sensitivity in rice germplasms

**Fig. 1:**
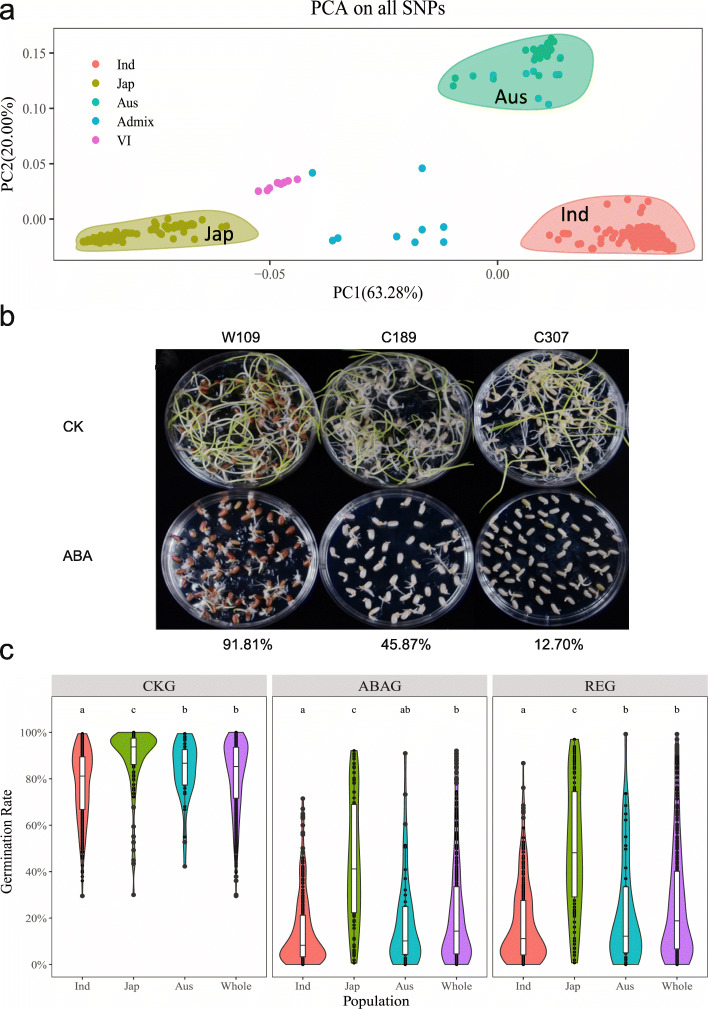
Population structure analysis and seed germination rate variation within the subpopulations and whole population. **a** Principal component (PC) analysis for all 425 accessions, including *indica* (Ind), *japonica* (Jap), *Aus* (Aus), and some intermediate (*VI and Admix*). **b** Some representative images of ABA inhibited seed germination phenotypes and corresponding REG. **c** Variation and statistical analysis for seed germination rate of CK group (CKG) and ABA-treated group (ABAG), and the relative germination rate (REG, ABAG/CKG × 100%) in the subpopulations and whole population

Considering that the population size in this study (425 accessions) was smaller than the original population (529 accessions) used for GWAS (Chen et al. 2014), a principal component (PC) analysis based on 4.3 million SNPs was conducted to examine the population structure. The results showed that *indica* (263 accessions), *japonica* (104 accessions), and *Aus* (42 accessions) subpopulations were separated by PC1 (explaining 63.28% of the total genetic variation) and PC2 (explaining 20.00% of the total genetic variation) (Fig. 1a**)**. This population structure was essentially the same as that of the original population.

Seed germination inhibited by ABA has been commonly used as criteria to evaluate ABA sensitivity. To investigate the genetic variation of ABA sensitivity in the rice population, matured and dried seeds were used for germination with or without ABA treatment. Seed germination percentage under non-treatment conditions (CKG) and ABA-treated conditions (ABAG) as well as relative germination percentage (REG), ratio of ABAG/CKG, were investigated in the whole population containing all 425 rice accessions, and the three major subpopulations. Some representative images of ABA inhibited seed germination phenotypes and corresponding REG are shown in Fig. 1b. CKG in *indica*, *japonica*, *Aus*, and the whole population ranged in 29.51%–99.44%, 29.98%–100%, 42.25%–99.46%, and 29.51%–100%, respectively, thus indicating that large variations in seed germination existed in rice germplasms (Fig. 1c). ABAG in *indica*, *japonica*, *Aus*, and the whole population varied in the range of 0.00%–71.48%, 0.06% − 92.13%, 0%–90.98%, and 0%–92.13%, respectively. The mean seed germination rate under ABA treatment was significantly lower than that in the non-treatment control (*P* < 0.001), suggesting that the inhibition of seed germination by ABA was effective and reliable in this experiment. The range of REG variations in *indica*, *japonica*, *Aus*, and the whole population were 0.00%–86.70%, 0.07%–96.98%, 0.00%–99.26%, and 0.00%–99.26%, respectively. These results clearly indicated there was a large genetic variation in ABA sensitivity in the subpopulations and the whole population. Among the whole populations, 11 *indica* accessions (C019, C020, C039, C069, C147, C159, C168, W058, W180, W187, W259) and 3 *Aus* accessions (W108, W131, W292) showed very strong hypersensitivity to ABA (REG < 0.50%), while 7 *japonica* accessions (C085, C111, C146, W053, W060, W109, W193), 1 *Aus* accession (W330) and 1 Admix accession (W097) showed no obvious sensitivity to ABA (REG > 90.00%) (Supplementary Table 1). Statistical analysis revealed significant difference in the seed germination ability among the subpopulations. The mean of CKG (88.52%), ABAG (45.77%) and REG (50.10%) in *japonica* were significantly higher than those in *indica* (77.26%, 14.52%, 17.77%, respectively) and *Aus* (83.47%, 18.98%, 22.94%, respectively) (*P* < 0.05, Fig. 1b). This result indicates that *japonica* varieties are generally weaker in seed dormancy and less sensitive to ABA than *indica* and *Aus* varieties.

### Association mapping of ABAG and REG

**Fig. 2:**
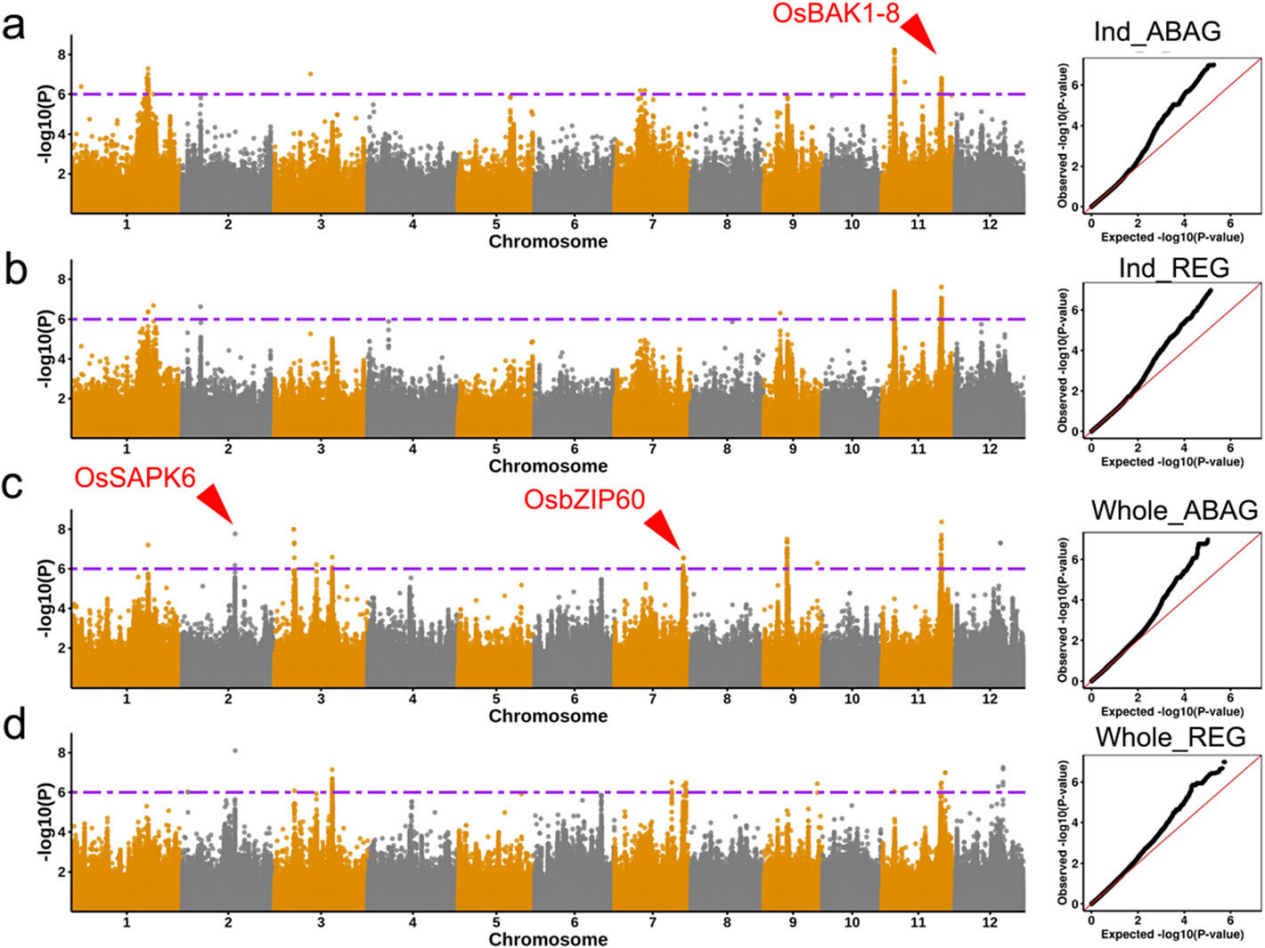
GWAS for seed germination rate in the *indica* subpopulation and whole population. Manhattan and Quantile-quantile plots are shown for ABAG in the *indica* (**a**), REG in the *indica* (**b**), ABAG in the whole populations (**c**), and REG in the whole population (**d**) using FaST-LMM. Red arrows indicate the genes are considered to be important candidates related to ABA sensitivity. Dashed horizontal line for each population indicates the suggestive threshold (*P* = 1.0 × 10^− 6^)

Since the seed germination ability varied under normal germination conditions, REG under ABA treatment was also used for association mapping of ABA sensitivity in addition to ABAG. Factored Spectrally Transformed Linear Mixed Model (FaST-LMM) was used to identify association signals in the *indica*, *japonica* and whole populations, using a total of 4.3 million SNPs in the 425 rice accessions. Several significant association signals were detected in the whole population and the *indica* subpopulation, as indicated by Manhattan and Quantile-quantile plots (Fig. 2). However, no significant signal was detected in the *japonica* subpopulation. Although marginally significant SNPs were detected for ABAG or REG in the *Aus* subpopulation, they were not subjected for further analyses because of small population size.

A total of 131 and 188 SNPs for ABAG and 100 and 67 SNPs for REG were detected by GWAS in the *indica* subpopulation and the whole population, respectively, at a significance level of -log (*P*) > 6. Based on reported genome-wide linkage disequilibrium (LD) decay distance (Huang et al. 2010; Huang et al. 2012), all variants in high LD (*r*^2^ > 0.6) of significant SNPs within a range of 150 kb were merged as an association locus. Adjacent association loci were merged into single association locus when they were overlapped. The association loci with a length of shorter than 5 kb were ignored.

In the *indica* subpopulation, 17 and 12 association loci were detected for ABAG and REG, respectively. In the whole population, 22 and 18 association loci were detected for ABAG and REG, respectively. A total of 48 non-redundant loci were detected for ABAG or REG in, at least, one population (Supplementary Table 2). These results suggest that genetic variations of ABA sensitivity may be controlled by multiple loci in rice.

### Genes related to ABA or stress responses within the significant loci of REG and ABAG

**Fig. 3:**
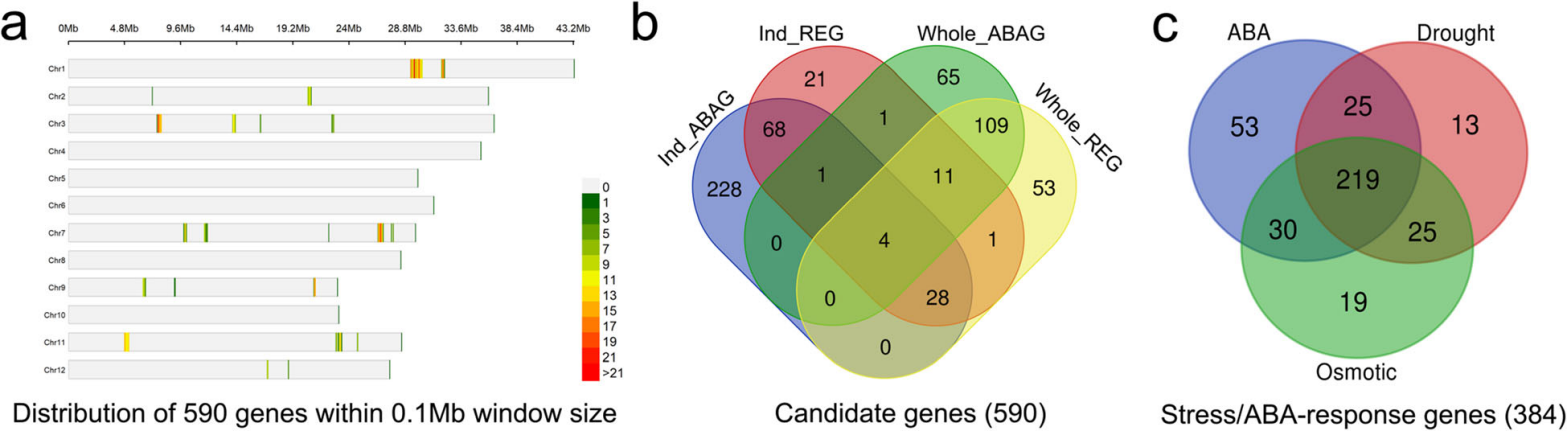
Gene analysis within the association loci. **a**. Chromosomal distribution of 590 genes in the loci associated with ABA sensitivity. Color scale indicates the number of genes in the associated loci. **b**. Venn diagram showing the distribution of the genes in the associated loci for ABAG in the *indica* subpopulations (Ind_ABAG) and whole population (Whole_ABAG), and the genes in the associated loci for REG in the *indica* subpopulations (Ind_REG) and whole population (Whole_REG). **c**. Venn diagram showing the number of the genes from the association loci are responsive (threshold of 2-fold-change) to ABA, drought, and/or osmotic stresses. The relative expression levels of these genes in the treatment of ABA and drought or osmotic stress were extracted from a public database (https://tenor.dna.affrc.go.jp)

In order to identify genes involved in ABA sensitivity, all the non-transposon genes in the association loci of REG and ABAG were extracted from the annotated Nipponbare reference genome. A total of 590 non-redundant and non-transposon genes were annotated in the 48 non-redundant association loci distributed in chromosome 1, 2, 3, 7, 9, 11, and 12 (Fig. 3a, Supplementary Table 3). There were 329 and 135 genes in ABAG and REG association loci, respectively, in the *indica* subpopulation, and 191 and 206 genes in ABAG and REG association loci, respectively, in the whole population (Fig. 3b). In total, 221 genes were identified in the common loci for ABAG and REG. Among them, 4 genes including LOC_Os11g39330, LOC_Os11g39350, LOC_Os11g39360, and LOC_Os11g39370 (*OsBAK1–8*) were presented in the loci for ABAG and REG in both the *indica* subpopulation and the whole population.

By searching the expression levels of 590 genes in a public database (https://tenor.dna.affrc.go.jp), a total of 386 non-redundant genes were found to be responsive to ABA or stresses. Among them, 327, 282, and 293 genes were responsive to ABA, drought, and osmotic stress, respectively, and 219 genes were responsive to all the three treatments (Fig. 3c). These results suggest that ABA and stress-responsive genes are highly enriched in the loci for ABA sensitivity.

According to the function annotation and/or reported functions of the genes in the significantly associated loci for ABAG and REG, at least nine genes, including *OsSAPK6* (LOC_Os02g34600) (Saha et al. 2014), *bZIP60* (LOC_Os07g44950) (Xu et al. 2011), *OsBAK1–8* (LOC_Os11g39370) (Khew et al. 2015), *OsCIPK30* (LOC_Os01g55440) (Kanwar et al. 2014), *OsCIPK12* (LOC_Os01g55450) (Kanwar et al. 2014), *OsCBL10* (LOC_Os01g51420) (Kanwar et al. 2014), *OsFUS3* (LOC_Os01g51610) (Brocard-Gifford et al. 2003), and *OsBPM* (LOC_Os07g20130) (Julian et al. 2019), were directly or indirectly related to ABA signaling and its regulation.

### Haplotype analysis of selected candidate genes

**Fig. 4:**
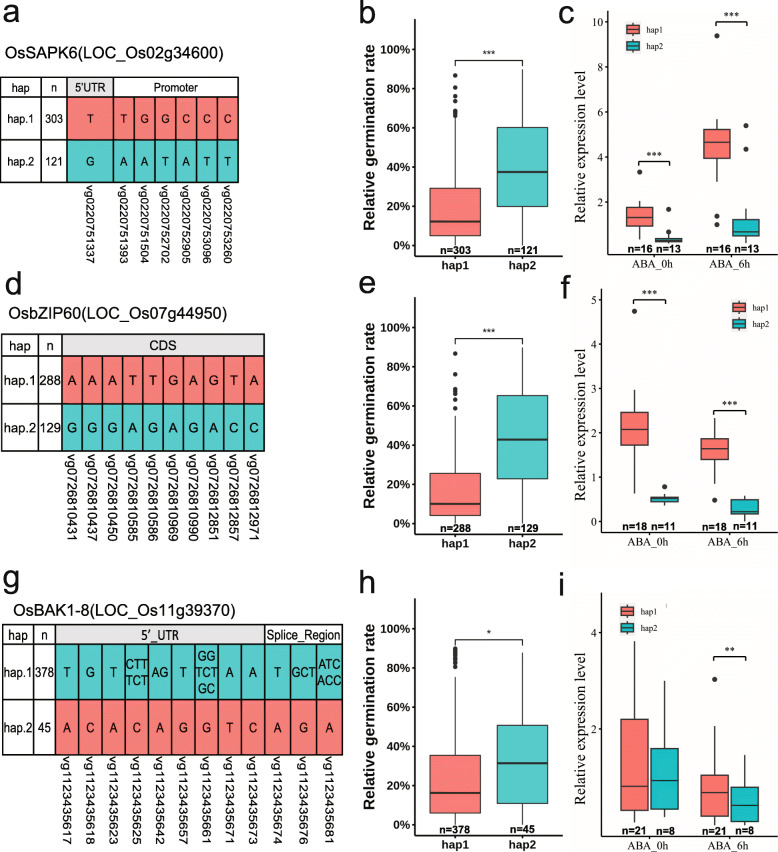
Haplotype and expression level analysis of the three candidate genes. Locations of key SNPs used for haplotype analysis are shown for the three genes including *OsSAPK6* (LOC_Os02g34600, **a**), *OsbZIP60* (LOC_Os07g44950, **d**), and *OsBAK1–8* (LOC_Os11g39370, **g**). The REG of hap1 (303 accessions) and hap2 (121 accessions) in *OsSAPK6* (**b**), hap1 (288 accessions) and hap2 (129 accessions) in *OsbZIP60* (**e**), hap1 (387 accessions) and hap2 (45 accessions) in and *OsBAK1–8* (**h**) in the whole population are shown. The relative expression levels of hap1 group (16 accessions) and hap2 group (13 accessions) in *OsSAPK6* (**c**), hap1 group (18 accessions) and hap2 group (11 accessions) in *OsbZIP60* (**f**), and hap1 group (21 accessions) and hap2 group (8 accessions) in *OsBAK1–8* (**i**) in response to ABA at 0 h and 6 h are presented

To further test which of the ABA signaling-related candidate genes in the association loci were involved in ABA sensitivity, all SNPs or InDels in the candidate genes were extracted from RiceVarMap 2.0 (Zhao et al. 2015) for haplotype analysis. All non-synonymous SNPs/InDels within the coding regions and the most significantly associated SNPs/InDels in promoter regions were selected for haplotype analysis. Among the ABA and stress-responsive genes in the loci associated with REG or ABAG, haplotype analysis results of three candidate genes including *OsSAPK6*, *bZIP60*, and *OsBAK1–8* were presented in Fig. 4 and described as follows. The information of key SNPs and InDels in each candidate gene was shown in Supplementary Table 4.

OsSAPK6 is a member of SnRK2 protein kinase family in the ABA signaling core (Saha et al. 2014). This gene is responsive to drought, osmotic stress, and ABA treatment (Supplemental Fig. 1). Seven SNPs in *OsSAPK6* were considered as possible causal variations between haplotype 1 (hap1) and haplotype 2 (hap2) (Fig. 4a). Two SNPs (vg0220751393 and vg0220751504) were located in two putative AP2/ERF-binding elements. Two SNPs (vg0220751393 and vg0220752702) were located in putative NF-YA/YB/YC elements. Vg0220751504, vg0220753260 and vg0220753096 were located in a putative MYB-binding element, a putative bZIP-binding element, and a putative ABA-responsive element (ABRE), respectively. REG was significantly different between hap1 (15.5%, 303 accessions) and hap2 (30.5%, 121 accessions) in the whole population (*P* < 0.001, Fig. 4b). These results indicate that the expression of *OsSAPK6* might be affected by these SNPs. We performed qPCR to detect the expression levels of *OsSAPK6* in seedling leaves with or without ABA treatment in randomly selected ABA-insensitive and ABA-sensitive rice accessions. These accessions were divided into two groups to compare the expression levels of *OsSAPK6*. The hap1 group (16 accessions) had significantly higher expression levels of *OsSAPK6* than hap2 group (13 accessions) under both normal and ABA treatment conditions, and the difference under ABA treatment was more obvious (Fig. 4c). This result indicates that the difference in the expression levels of *OsSAPK6* between the two haplotypes might contribute to the difference in ABA sensitivity.

*OsbZIP60* was up-regulated by heat and drought stresses as well as ABA treatment (Supplemental Fig. 1). Ten non-synonymous SNPs in the coding region of *OsbZIP60* caused amino acid changes including Gly172Ser, Ala174Thr, Gly178Asp, Gln223Leu, Gln223His, Glu351Gly, Gly358Glu, Gly484Asp, Thr486Ile, and Ala524Glu between two haplotypes (Fig. 4d). Among them, Gly172Ser occurred in bZIP domain, and it may affect the function of OsbZIP60. There was also a significant difference in REG between the two haplotypes of *OsbZIP60* in the whole population (*P* < 0.001, Fig. 4e). Although there were no significant variations detected in the *OsbZIP60* promoter, the expression levels showed a significant difference between the two haplotypes (16 accessions of hap1 and 11 accessions of hap2) (Fig. 4f). These results suggest that, in addition to the coding variations of *OsbZIP60*, other distal regulatory elements may be involved in the function of this gene in the regulation of ABA sensitivity.

*OsBAK1–8* encodes a homologue of Arabidopsis BAK1, which is responsive to ABA and can interact with SnRK2.6 (Khew et al. 2015; Shang et al. 2016). *OsBAK1–8* is slightly responsive to drought stress and ABA treatment (Supplemental Fig. 1). The two major haplotypes were identified for *OsBAK1–8* with seven SNPs and five InDels (Fig. 4g). There was also a significant difference in REG between the two haplotypes in the whole population (*P* < 0.05, Fig. 4h). An important InDel (vg1123435674) located at the splice acceptor sequence of GT-AG can generate different open reading frames, which may affect the function *OsBAK1–8* related to ABA sensitivity. It cannot be excluded that the SNPs in 5’UTR may affect the expression level of *OsBAK1–8.* However, qPCR analysis showed that, unlike the former two candidate genes, there was no difference in the expression levels of *OsBAK1–8* between the two haplotypes (21 accessions of hap1 and 8 accessions of hap2) (Fig. 4i). This result suggests that further studies may focus on the variation at splicing site of *OsBAK1–8* to reveal its relationship with ABA sensitivity in rice.

## Discussion

Although the ABA signaling core has been intensively studied and widely accepted, the genetic variation of ABA signaling and its physiological “output” (collectively called ABA sensitivity) remain largely unknown. Therefore, identification of more genetic components of ABA sensitivity is critical to illustrate how ABA signaling fine tunes the plant responses to environmental cues and stresses. In this study, we investigated the genetic variation of ABA sensitivity (in terms of the seed germination capability under ABA treatment) for the first time in a large rice panel containing 425 accessions worldwide. We observed that the seed germination capability without ABA treatment dramatically varied in the *indica* subpopulation, but less variation was observed in the *japonica* subpopulation. Similar result has been reported in an early study on rice seed dormancy (Magwa et al. 2016). Our results also showed that the *indica* or *Aus* subpopulation were generally more sensitive to ABA than the *japonica* subpopulation. In accordance with the variation difference in ABA sensitivity, GWAS detected 17 association loci for ABAG and 12 association loci for REG in the *indica* subpopulation, but failed to detect significant association loci in the *japonica* subspecies. However, it cannot be excluded that minor effective QTLs may be detected in the *japonica* subspecies if lower concentration of ABA is used in the treatment.

Within the 48 non-redundant association loci (an average size of 98 kb) detected by GWAS of ABAG and REG (Supplementary Table 2), 590 non-transposon genes were further analyzed for their candidacy related to ABA signaling and/or metabolism (Supplementary Table 3). Surprisingly, very few genes in these loci matched the expected functions in the ABA signaling or metabolism pathways. The PYL-PP2C-SnRK2-ABREB (ABI5/bZIP) module has been well recognized in the core ABA signaling pathway. The rice genome contains 12 OsPYLs (He et al. 2014; Kim et al. 2012), 10 clade A PP2Cs, 10 SnRK2 protein kinases family (SAPKs), and 11 bZIP transcription factors in subgroup A with functions similar to ABI5 (Zong et al. 2016). It was expected that some of these genes in the ABA signaling core might have significant variations associated with ABA sensitivity. However, according to our results, only *OsSAPK6* was detected with an associations with ABAG and REG in the whole population. One possibility could be that the functions of different members in the core ABA signaling gene families have been differentiated in the ancestral species of cultivated rice to fine tune not only ABA signaling but also other processes in plant growth and adaptation to various environmental cues, and therefore most of these core genes have no or marginal natural variations in ABA sensitivity in terms of the seed germination inhibition by ABA. In fact, only a few important natural variations have been reported for the core genes of other phytohormone signaling pathways, and the variations controlling many important agronomic traits occurred mainly in the regulatory or downstream genes of core signaling pathways. Another possibility could be that most of the germplasms or varieties used in this study are lowland or irrigated rice that have been domesticated or selected under non-stressed conditions, and therefore the core ABA signaling genes related to stress tolerance may be not necessary to be selected in lowland rice, unlike other upland crops such as maize (Shikha et al. 2017; Xiang et al. 2017).

OsSAPK6, a component in the ABA signaling core, has been found to be associated with ABA sensitivity. Previous studies have reported that OsSAPK6 is ABA-inducible and it is related to osmotic stress signaling (Chae et al. 2007; Kobayashi et al. 2004, 2005). OsSAPK6 can interact with and phosphorylate OsbZIP10 in response to ABA signaling in rice (Chae et al. 2007). OsSAPK6, as well as OsSAPK2 and OsSAPK9, can also phosphorylate and activate OsbZIP46 to pass on ABA signaling to gene expression (Kim et al. 2015; Tang et al. 2012). Our results showed that most of the significantly associated SNPs in *OsSAPK6* were located in its promoter or 5’UTR region, and the expression levels of *OsSAPK6* were higher in hap1 than that in hap2, indicating that the expression levels of *OsSAPK6* might vary in rice germplasms, thereby affecting ABA sensitivity. In fact, the two haplotypes of *OsSAPK6* based on the significant SNPs in the promoter showed significant difference in both ABA sensitivity and the expression level of the gene itself. Our previous study showed that overexpression of *OsSAPK6* enhanced ABA sensitivity as well as drought and heat tolerance (Chang et al. 2017), which also supported the important role of transcriptional regulation of *OsSAPK6* in ABA signaling. The significant variations in the promoter may be targets for further identification of transcription factors regulating *OsSPAK6* expression or for designing molecular marker of superior allele for breeding selection.

In addition to *OsSAPK6*, at least eight genes with putative functions in regulating or amplifying ABA signaling were detected with significant associations with ABA sensitivity. LOC_Os01g51610 is a FUS3 homologue closely related to ABI3/VP1 which regulates seed germination by interacting with ABI4 and ABI5 (Brocard-Gifford et al. 2003). LOC_Os07g20130 is annotated as a homolog of BPM which regulates seed germination by affecting degradation of PP2Cs (Julian et al. 2019), and it was reported to be up-regulated by heat and drought stresses (Xu et al. 2011) as well as ABA treatment (https://tenor.dna.affrc.go.jp). The haplotype analysis suggested that the variations in the coding region of *OsbZIP60* may mainly contribute to the function of this gene in ABA response. Nevertheless, other distal regulatory elements may be also involved in the regulation of *OsbZIP60* since the two haplotypes showed difference in the expression levels of this gene under ABA treatment. OsBAK1–8, a homologue of BAK1, might regulate seed germination by affecting the activity of SnRK2 (Khew et al. 2015). It was reported that BAK1 can form a complex with OST1 (SnRK2.6 or SRK2E) near the plasma membrane and that ABA can enhance the formation of the complex in Arabidopsis (Shang et al. 2016). The haplotype analysis and qPCR analysis suggested that the variation at a splicing site of *OsBAK1–8* might be an important clue to track down the causal variation of this gene for its contribution to ABA sensitivity. The haplotype and expression level analyses of these genes provided additional evidence for their roles in ABA sensitivity, and further genetic and molecular studies of these candidate genes will uncover their exact functions related to ABA signaling or regulation. It should be noted that there are some reported cases in which the functional variants are large structure variations (Yuan et al. 2021), however the structure variations cannot be detected in the dataset used in this study. Large structure variations can be further excluded or confirmed by re-sequencing the genomic sequences covering the coding and regulatory regions of the important candidate genes in a condensed panel of the population or by waiting for the update of RiceVarMap with large structure variations included in the future.

It is interesting to notice that three calcium signaling-related genes, including *OsCIPK30*, *OsCIPK12* and *OsCBL10*, were detected with significant associations with ABA sensitivity. In Arabidopsis, several CIPKs and CBLs have been reported to regulate ABA signaling. For example, CIPK11 phosphorylated ABI5 and mutation of the phosphorylation site in *ABI5* resulted in higher trans-activation activity of ABI5 (Zhou et al. 2015). CBL2 might be essential for appropriate ABA responses because seed germination in the *cbl2* mutant was hypersensitive to ABA (Batistic et al. 2012). CBL9 and CIPK3 can interact with each other to regulate the ABA responses in seed germination (Pandey et al. 2008). It will be intriguing to reveal whether and how *OsCIPK30*, *OsCIPK12* and *OsCBL10* regulate ABA responses in rice.

ABA plays a crucial role in plant adaptation to adverse environmental stresses. In our previous study, drought resistance association loci were detected by GWAS in rice using a large population containing the accessions used the current study (Guo et al. 2018). Of these loci, seven lead SNPs were located in ABA sensitivity loci in this study. One of them was located in the same ABA sensitivity locus containing *OsSAPK6* (Supplementary Table 2). We also found that two ABA sensitivity loci were overlapped with yield QTLs under drought stress conditions (Bhandari et al. 2020) (Supplementary Table 2). One of the loci also contained *OsSAPK6* gene. However, no significant correlation was found between ABA sensitivity and drought resistance in the population used in this study (data not shown), suggesting that ABA sensitivity in terms of seed germination inhibition by ABA cannot completely reflect drought resistance because the latter is more complex and involves multiple processes at different growth and developmental stages. Nevertheless, the co-localization of several ABA sensitivity loci with drought resistance QTLs, such as the locus containing *OsSAPK6*, suggests that ABA sensitivity might be closely related to drought resistance. In addition, the expression data from TENOR database (https://tenor.dna.affrc.go.jp) showed that among 590 non-redundant genes in the ABA sensitivity loci, 327 (55.42%), 282 (47.80%), and 293 (49.66%) genes were responsive to ABA, drought stress, and osmotic stress, respectively, and among these stress- or ABA-responsive genes, 57.03% (219 genes) were responsive to all the three treatments. Taken together, the partial overlapping of genetic loci for ABA sensitivity and drought resistance and the largely enriched stress-responsive genes in the ABA sensitivity loci suggest that drought resistance in rice is closely related to ABA response but distinct ABA-independent mechanism may also exist, which has been well recognized in Arabidopsis (Soma et al. 2021).

## Conclusions

Genetic control of ABA sensitivity at seed germination was analyzed by genome-wide association mapping based on high density SNPs using 425 rice accessions. Totally, 48 non-redundant association loci were detected by GWAS of ABAG and REG in the *indica* or whole population. Eight loci of ABA sensitivity were overlapped with reported QTLs of drought resistance, one of which contained *OsSAPK6*, a component in ABA signaling core. This study provided new insights into the genetic basis of ABA sensitivity related to stress responses in rice, and the candidate genes identified in this study may be further explored for the genetic improvement of stress resistance.

## Methods

### Plant materials

An association population containing 529 rice accessions collected worldwide (Chen et al. 2014) were grown in the experimental station of Huazhong Agricultural University at Wuhan in 2016. Among the population, 425 accessions with sufficiently matured seeds harvested under the same conditions were selected for seed germination experiments.

### Phenotyping ABA sensitivity

The harvested dry seeds were kept in a seed storage chamber with temperature at 20 °C and relative humidity below 10%. For germination evaluation, seeds of each accession were successively surface-sterilized with 70% ethanol for 2 min, 0.15% HgCl_2_ solution for 15 min, and then rinsed with sterile distilled water. Fifty sterilized seeds were transferred into plates (9 cm petri dish) with half-strength Murashige and Skoog (1/2 MS) medium for the control check (CK) group or 1/2 MS medium with 2 μM ABA for the treatment group. Three replicates were arranged for each group. All the above-mentioned seeds in the medium were placed in a growth chamber (26 °C and 100% relative humidity) under dark conditions. A seed was considered germinated when its radicle or coleoptile reached a length of ≥2 mm. The percentage of germinated seeds in each plate was recorded on the 7th day after seeding. The mean values of three replicates of germination rates of CK (CKG) and ABA treatment group (ABAG) were used for association analysis. Relative germination rate (REG), calculated as (ABAG /CKG) × 100%, was also used for association analysis because large variation in CKG exists in rice germplasms.

### Association mapping

The genotypic data of the association population generated by next generation sequencing was collected from a previous study (Chen et al. 2014). The population structure was constructed by a random effect in linear mixed model (LMM) using the kinship (K) matrix, and GWAS was performed using LMM provided by the FaST-LMM programme (Lippert et al. 2011). The numbers of SNPs used for GWAS for the whole population, *indica* and *japonica* subpopulation were 3,916,415, 2,767,159 and 1,857,845, respectively. All these SNPs in the whole population were used for principal component (PC) analysis by PLINK (Purcell et al. 2007). As some SNPs were closely linked, effective numbers of independent SNPs were determined by PLINK (window size 50, step size 50, *r*^*2*^ ≥ 0.2). Suggestive threshold was calculated based on the formula -log10 (1/effective number of independent SNPs) (Yang et al. 2014).

### Haplotype analysis

The genomic DNA sequences of candidate genes were used for haplotype analysis among the 425 accessions (Chen et al. 2014). The trait differences between alleles of each SNP in the candidate genes were assessed by Student’s *t*-tests. Among the significantly associated SNPs, the non-synonymous or other large effect SNPs/InDels within gene coding regions and SNPs/InDels in promoter regions with relatively lower *P* value were used for haplotype analysis.

### Expression level analysis

Total RNA was isolated from rice leaves, which were collected from two-week-old rice seedlings treated by 100 μmol ABA for 0 h or 6 h, by using TransZol reagent (TransGen Biotech, Beijing, China). RNA was reverse-transcribed by using EasyScript One-Step gDNA Removal and cDNA Synthesis SuperMix (TransGen Biotech, Beijing, China) according to the manufacturer’s instructions. Relative quantitative PCR (qPCR) was performed in an optical 384-well plate with an Applied Biosystems ViiA7 real-time PCR system (Applied Biosystems, USA) by using SYBR Premix Ex-Taq reagent (TaKaRa, Tokyo, Japan). The qPCR was conducted with the rice ubiquitin gene as an internal control.

## Supplementary Information

Additional file 1: Supplementary Table 1. Accession names and trait values (the mean of three replicates) for CKG, ABAG, and REG.

Additional file 2: Supplementary Table 2. Association loci of ABAG and REG in the *indica* subpopulation and whole population.

Additional file 3: Supplementary Table 3. Genes information in all association loci.

Additional file 4: Supplementary Table 4. Key SNPs and InDels in the three candidate genes.

Additional file 5: Supplementary Fig. 1. The relative expression levels of the three candidate genes under different stresses.

## Data Availability

All data and materials are available in the paper and online supplemental files.
