# Supplementary material for: Genome-wide association study revealed genetic variations of ABA sensitivity controlled by multiple stress-related genes in rice
Source: Stress Biol. 2021 Oct 7;1(1):10. doi: 10.1007/s44154-021-00011-4 (PMC10441979; doi:10.1007/s44154-021-00011-4)
Supplement: Supplementary file 5 — Supplementary Fig. 1. The relative expression levels of the three candidate genes under different stresses. [file 44154_2021_11_MOESM5_ESM.pdf]

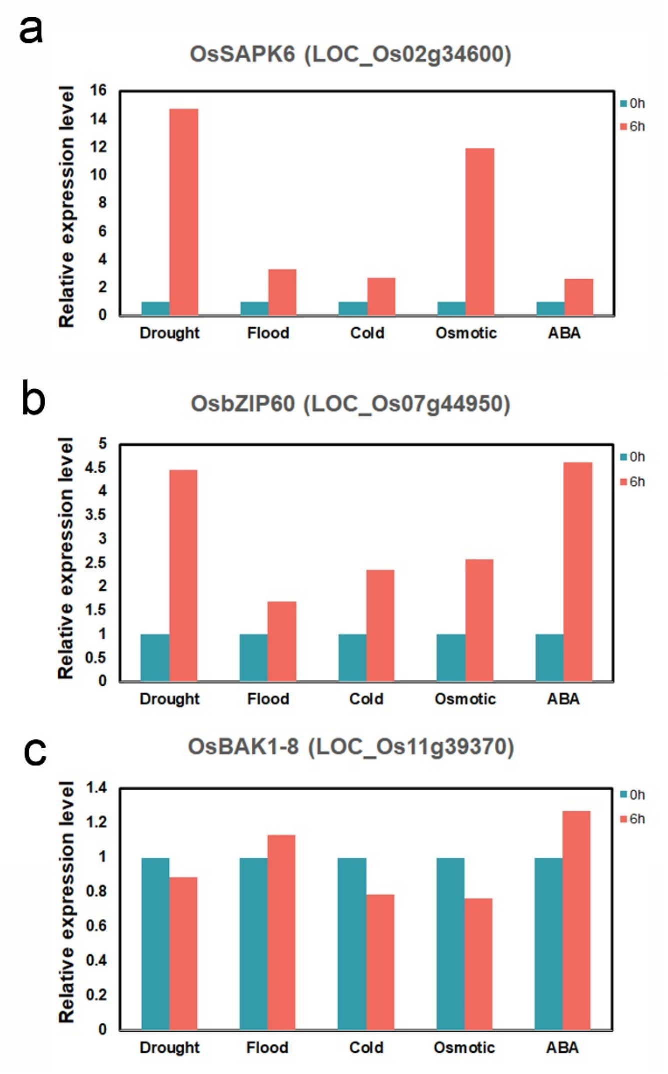

Supplementary Figure 1 Relative expression levels of three candidate genes under different stresses.

The relative expression levels of *OsSAPK6* (a), *OsbZIP60* (b), and *OsBAK1-8* (c) in response to drought, flood, cold, osmotic and ABA stress (Os) at 0 h and 12 h were presented based on the original data from public database TENOR (https://tenor.dna.affrc.go.jp).
